# Lattice Ultrasensitivity Amplifies Signals in *E. coli* without Finely-Tuned Allosteric Interactions

**DOI:** 10.1103/7dgz-l5fd

**Published:** 2026-06-08

**Authors:** Derek M. Sherry, Isabella R. Graf, Samuel J. Bryant, Thierry Emonet, Benjamin B. Machta

**Affiliations:** 1 Department of Physics, Yale University, New Haven, Connecticut 06511, USA; 2 Quantitative Biology Institute, Yale University, New Haven, Connecticut 06511, USA; 3 Developmental Biology Unit, European Molecular Biology Laboratory, 69117 Heidelberg, Germany; 4 Department of Physics and Astronomy, Heidelberg University, 69120 Heidelberg, Germany; 5 Department of Molecular, Cellular and Developmental Biology, Yale University, New Haven, Connecticut 06511, USA

## Abstract

The *E. coli* chemosensory lattice, consisting of receptors, kinases, and adaptor proteins, is an important test case for biochemical signal processing. Kinase output is characterized by precise adaptation to a wide range of background ligand levels and large gain in response to small relative changes in concentration. Existing models of this lattice achieve their gain through allosteric interactions between either receptors or core units of receptors and kinases. Here we introduce a model which operates through an entirely different mechanism in which receptors gate inherently far from equilibrium enzymatic reactions between neighboring kinases. Our lattice model achieves gain through a mechanism more closely related to zero-order ultrasensitivity than to allostery. Thus, we call it lattice ultrasensitivity (LU). Unlike other lattice models with critical points, the LU model can achieve arbitrarily high gain through timescale separation, rather than through finely tuned allosteric interactions. The model also captures qualitative experimental results which are difficult to reconcile with existing models. We discuss possible implementations in the lattice’s baseplate where long flexible linkers could potentially mediate interactions between neighboring core units.

## I. INTRODUCTION

The chemotaxis signaling pathway used by *E. coli* for run and tumble navigation is perhaps the best understood biological circuit. Predictive models can accurately describe how the sensory output—activity of the kinase CheA—responds to arbitrary time-varying inputs of ligand concentration [1–4] either in bulk experiments [5,6] or in individual cells [7–11]. Striking features of the response are large gain and precise adaptation over a wide range of background concentrations; following a small step change in attractant concentration, CheA activity responds in a fast, highly amplified manner (large gain). Methyltransferase CheR and methylesterase CheB then methylate and demethylate receptors, slowly adapting the system output back to its original, steady-state level (precise adaptation). The proteins that implement these dynamics are well characterized, and their structures have been resolved at atomic resolution [12–14]. The minimal unit capable of responding to signals is called the core signaling unit (CSU), comprising one CheA homodimer, two trimers of transmembrane receptor dimers, and two copies of a structural protein called CheW [15,16]. Neighboring CheAs and CheWs associate, connecting adjacent CSUs and forming the baseplate of a relatively stable lattice [17–19].

Established models of chemosensing, such as Monod-Wyman-Changeux (MWC) or Ising models, treat receptors as two-state systems (or four-state systems with binding and conformational state separated) which determine the activity of their associated kinases [1–3]. In these models equilibrium allosteric coupling between receptors in the lattice allows for amplification of small signals while methylation allows for adaptation by shifting the relative free energies of the active and inactive state. While these models successfully capture the average response of signaling to time-varying ligand, they are hard to reconcile with some results. First, recordings from individual cells show enormous fluctations [7,8,20] and even coordinated two-state switching when receptors are homogeneous [21], strongly suggesting a model in which kinases are coupled near a critical point of a lattice model [21,22]. Second, experiments explicitly measuring ligand binding found smaller gain and a smaller methylation induced shift in ligand sensitivity relative to kinase activity [23,24], inconsistent with equilibrium coupling between receptors and kinases and suggesting a nonequilibrium coupling between them [25]. Third, a reduction in gain and methylation dependence is observed in kinase activity in cells with a CheW mutation that prevents individual CSUs from combining into lattices [26]. While distinct existing models capture some of these qualitative features, we are unaware of any existing model which captures all three.

Here we introduce a model where nonequilibrium chemical reactions *between* neighboring CSUs implement gain. When CSUs are incorporated into lattices, these far from equilibrium reactions naturally enhance the methylation dependence and gain in kinase activity when compared to isolated CSUs. These reactions, facilitated by energy released during ATP hydrolysis, could be mediated by the long flexible linker connecting the phosphoaccepting P1 domain to the rest of the kinase. The gain in our model is mathematically equivalent to the zero-order ultrasensitivity described by Goldbeter and Koshland, wherein the concentration of the product of an enzymatic futile cycle depends sharply on the ratio of the rates of the forward and backwards enzyme reactions [27]. For this reason, we call our model the lattice ultrasensitivity (LU) model.

The LU model can capture experimental dose response curves similarly to classic models while also reproducing the large noise and two-state switching seen in measurements of single cell kinase activity [7,8]. As with recent nonequilibrium models [22,25], the LU model captures the differences in methylation dependence and gain between kinase activity and receptor binding assays. However, it also explains the reduced gain and methylation dependence in isolated CSUs.

The LU model is also appealing from an evolutionary design perspective. Both the LU model and the Ising model achieve large gain through proximity to a critical point, each requiring two parameters to be near their critical value. In each model, methylation dynamics tune one of these parameters. In the Ising model the other parameter (J) is an allosteric interaction which must be tuned near a critical value by evolution. By contrast, in the LU model the second parameter is a kinetic rate which is zero at the critical point. As a result, large gain can be achieved simply by having that reaction be very slow, without the fine tuning of allosteric interactions required of all past lattice models.

Finally, the key nonequilibrium reactions in the LU model present a remarkably different picture of the mechanism behind chemotaxis signaling. Our model is reminiscent of modern treatments of signal transduction in eukaryotes, where many intermediate processing steps are driven by irreversible chemical reactions like phosphorylation, rather than by equilibrium allostery, and where flexible disordered linkers on otherwise structured proteins mediate interactions even in densely packed assemblies [28].

## II. MODEL AND RESULTS

### A. Signaling in individual CSUs is enhanced through nonequilibrium coupling

**FIG. 1.**
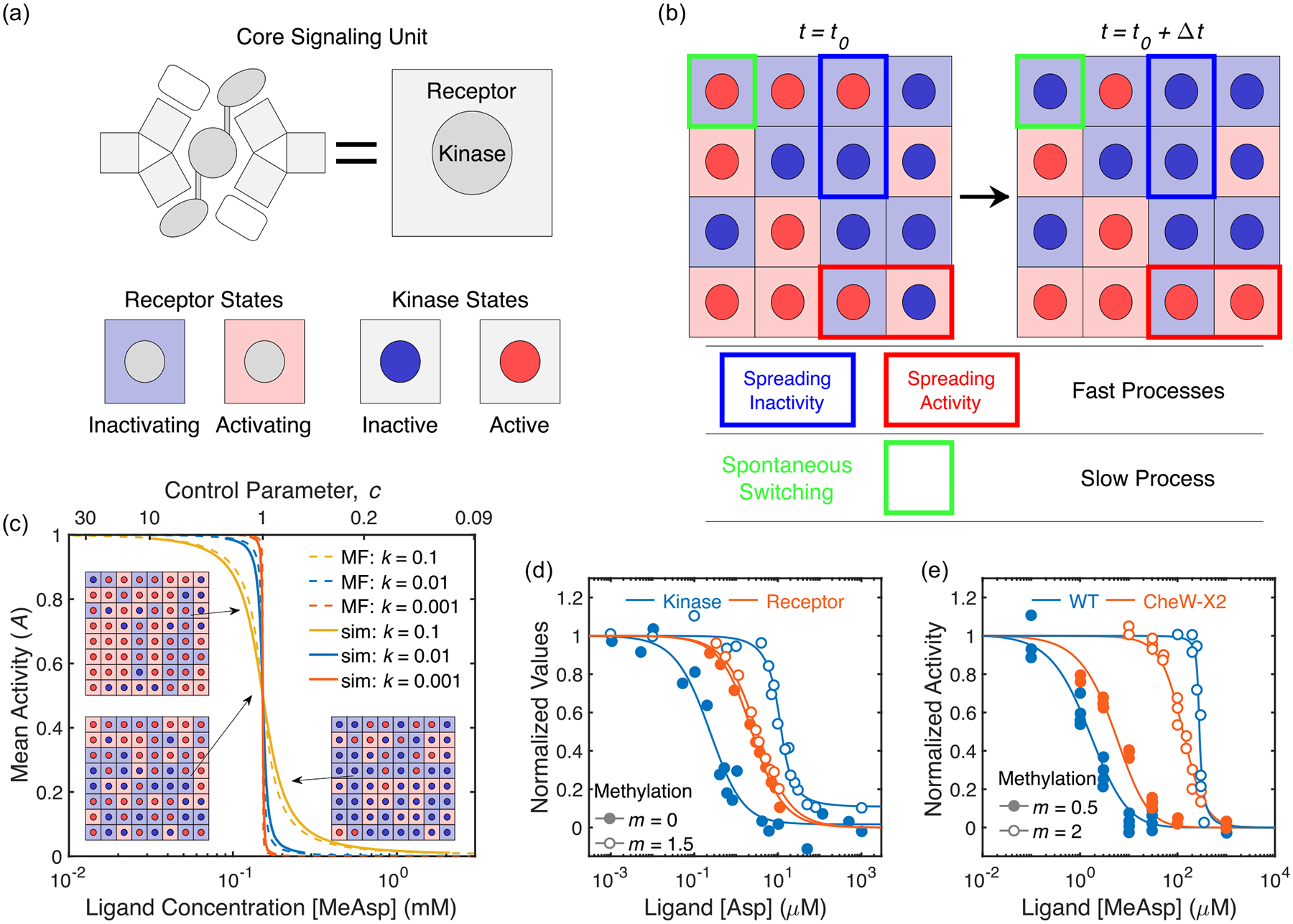
Overview of Lattice Ultrasensitivity. (a) CheA kinases are represented by circles, whose color indicates their activity state (active or inactive). Receptors are represented by the surrounding squares, and their color indicates a preference for spreading of activity vs inactivity. (b) Examples of spreading and spontaneous switching. The two configurations show one realization of each process occurring on a lattice in a time Δt. Colored rectangular outlines highlight the core units impacted by the processes indicated below. The probability of spreading activity or inactivity depends on the control parameter c, defined as the ratio of total rates of kinase activation and deactivation, which themselves depend on receptor occupancy and methylation [Eq. (4)]. In this case c=1. (c) The cooperativity of dose response curves increases as k becomes small. Mean-field predictions agree with simulations. Insets show representative lattice configurations for the indicated activity levels; note that the changes in kinases activity between insets are amplified relative to changes in receptors. Here we’ve chosen $m=m_{0s}$. (d) Fits to *in vitro* experimental data from Amin and Hazelbauer [24]. Nonequilibrium spreading allows for adaptation to be largely absent from receptor binding, but present in kinase activity. (e) Separate fits to *in vitro* experimental data from Frank *et al*.[26]. When the sensory lattice is disrupted by the CheW-X2 mutant, spreading is no longer possible. Adaptation and gain are reduced, similarly to receptor binding. Fit parameters for (d) and (e) are in Appendix A.

We begin with a description of an individual, isolated CSU, containing a single receptor cluster and one kinase homodimer [Fig. 1(a)]. This CSU is described by three dynamic variables corresponding to the receptor state, the kinase activity, and the methylation level.

In our model, receptor state describes a coarse graining of the low-affinity and high-affinity configurations of the ligand binding domains in one CSU. Similarly, the kinase state describes the kinase’s (assumed binary) ability to carry out phosphorylation reactions. How these coarse-grained states map onto configurations of proteins, and how they are coupled is the subject of ongoing research by many groups [12,29,30]. The binary kinase state in our model is a coarse-grained representation of the configuration of multiple kinase and receptor domains.

We model receptor state as an equilibrium MWC cluster with a free energy difference between kinase-inactivating and kinase-activating states:

(1) $$\Delta f([L],m)=-\alpha_{r}\left(m-m_{0r}\right)+\text{log}\left(\frac{1+[L]/K_{i}}{1+[L]/K_{a}}\right),$$

where $K_{i/a}$ are the ligand affinities of the two states, $\alpha_{r}$ parametrizes the dependence of the free energy on the methylation level m,[L] is the instantaneous ligand concentration, and $\alpha_{r}m_{0r}$ is the free energy difference in the absence of ligand and methylation (m=0 and [L]=0). The probability p that a receptor is activating is then given by a Boltzmann distribution over the two states

(2) $$p([L],m)={\left(1+e^{n\Delta f([L],m)}\right)}^{-1},$$

where n denotes the degree of cooperativity within the receptor cluster. In standard MWC models for chemosensory gain, adaptation and gain are fully parameterized by α and n, respectively. In the LU model, these describe only the far smaller gain and methylation dependence observed in ligand binding assays [23,24]. Further gain and methylation dependence occur due to lattice interactions described below.

The kinase state in a CSU can be active or inactive and spontaneously switches between these two states at a base rate kv/2. Here v is the rate of a spreading process defined below, and k sets the ratio of switching to spreading rates. Rather than directly controlling the kinase activity, the receptor states in our model gate transitions between activity states: a kinase can become active only if its associated receptor cluster is activating, and it can become inactive only if its associated receptor cluster is inactivating. No other dynamics are present in isolated CSUs. Thus, the average kinase activity exactly reflects the average state of the MWC-like receptors, and the activity of isolated CSUs exhibits the same reduced gain and methylation dependenced observed in receptor binding.

Enhanced gain and adaptation appear in the LU model only after CSUs are connected in an extended lattice where “spreading” reactions couple neighboring kinase states. For simplicity we consider a square lattice [Fig. 1(b)]. An active kinase can spread activity to a neighboring inactive kinase, but only if that neighbor’s receptor is activating. This activation occurs at a methylation-dependent rate $\frac{v}{4}\left(1+e^{g(m)}\right)$ per active kinase, where ν sets the base spreading rate independent of receptor state p and methylation level m. Similarly, an inactive kinase can inactivate its neighboring active kinases at a rate $\frac{v}{4}\left(1+e^{-g(m)}\right)$, but only if its neighbor’s receptor is inactivating. Thus, the receptor state p gates activity changes via spreading the same way they gate spontaneous switching. We take $g(m)=\alpha_{s}\left(m-m_{0s}\right)$, where α>0 quantifies how strongly methylation affects spreading and $m_{0s}$ is the methylation level at which activation and inactivation rates are equal. In our model, as in Refs. [22,25], methylation level modulates the rate of a kinetic process, perhaps by altering a free energy barrier. This is distinct from the mechanisms through which methylation level influences kinase activity in equilibrium MWC and Ising models. In those models methylation shifts an equilibrium free energy difference between active and inactive states.

We chose the specific form of methylation dependence so that the ratio of the rate of activation to deactivation is equal to $e^{g(m)}$. This choice is consistent with other models [2,3,6] where the linear g(m) is the free-energy difference between active and inactive states. It is also consistent with experimental dose response curves (Appendix A) and ensures that the initial response speed after adaptation has minimal dependence on ligand and methylation levels (Appendix C). Our form of g(m) implies that once adapted, activity dynamics are independent of ligand concentration. While these dynamics are faster than is experimentally accessible, qualitative results do not depend strongly on this specific form.

Taken together, the dynamics of all kinase and receptor states in the LU model are summarized as follows:


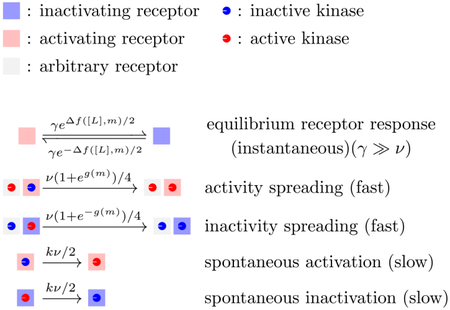


The spreading dynamics highlight the key distinguishing features of the LU model. In isolated CSUs, the distinct receptor and kinase states matter little because kinase activity exactly follows the receptor state. However, spreading interactions allow kinases to have strongly amplified and adapting responses even while receptors do not, as we show below.

Spreading is defined kinetically, and cannot be described using an equilibrium Hamiltonian. Spreading reactions occur at a rate proportional to the number of unlike neighbors, gated by the state of their receptor. These dynamics explicitly depend on the coarse-grained receptor state, but kinase dynamics have no direct reciprocal influence on the receptor state as they would in an equilibrium system. The kinase state influences the parts of the receptor conformation that control methylation and demethylation, but not those that control the ligand binding affinity (the receptor state p).

In addition, the linear dependence of spreading rates on the number of unlike neighbors is inconsistent with equilibrium dynamics and does not satisfy detailed balance. These dynamics are more similar to those of a noisy voter model [31] than to an Ising model. Furthermore, they suggest different underlying molecular mechanisms, driven by chemical reactions rather than thermally driven allosteric interactions.

Unlike the receptor and kinase dynamics, the methylation dynamics in the LU model follow the established mechanism for precise adaptation through integral feedback [32]. Each CSU has a methylation level between zero and four, reflecting the four possible methylation sites present on Tar, one of two main receptor types of *E. coli*. By only methylating receptors with inactive kinases and only demethylating receptors with active kinases, the average activity of kinases is guaranteed to robustly adapt to a specific, finite activity level, at least so long as most CSUs are not at an extreme methylation level. The adapted steady-state activity level is determined by the ratio of the methylation rate $k_{R}$ and the demethylation rate $k_{B}$.

In the following section, we use an analytical approximation to develop an intuition for the behavior of the LU model. Later we simulate dynamics of complete signaling lattices using a Gillespie algorithm [33] (Appendix B).

### B. Large gain arises by separating the timescales, k≪1

We begin our analysis of the LU model using an analytical mean-field theory which explicitly ignores spatial structure in the full system. In this approximation, the time evolution of the average kinase activity A is described by a simple differential equation:

(3) $$\frac{1}{v}\frac{\text{d}A}{\text{d}t}=p([L],m)(1-A)\left[\left(1+e^{g(m)}\right)A+2k\right]-\left[1-p\left(\left[L\right],m\right)\right]A\left[\left(1+e^{-g\left(m\right)}\right)\left(1-A\right)+2k\right].$$

The first term on the right-hand side is the rate at which inactive kinases become active via spreading and spontaneous switching, accounting for the restrictions placed on these processes by the receptor state. The second term similarly describes inactivation. We’ll use this equation to explain how large gain arises in the spreading dominated regime where k≪1, and why receptors and isolated CSUs show less methylation dependence than CSUs that are part of intact lattices.

We start by examining the dynamics in the absence of switching (k=0), where the entire right-hand side of Eq. (3) becomes proportional to A(1-A). The right-hand side is positive if the spreading activation rate is higher than the inactivation rate, namely, if $p([L],m)\left(1+e^{g(m)}\right)>(1-p([L],m))\left(1+e^{-g(m)}\right)$ and negative otherwise. We will call the ratio of these two spreading rates the control parameter,

(4) $$c(m,[L])=\frac{p([L],m)\left(1+e^{g(m)}\right)}{[1-p([L],m)]\left(1+e^{-g(m)}\right)}\;=e^{g(m)-n\Delta f([L],m)},$$

which is an increasing function of m and a decreasing function of [L]. In this k→0 limit, when c>1 activity increases until it reaches a stable fixed point at A=1. When c<1 activity decreases and the stable fixed point is at A=0. Thus, the steady-state activity level is $A^{*}=\Theta (c-1)$, where Θ is the Heaviside function. At c=1, the susceptibility dA/dc diverges and $A^{*}$ is extremely sensitive to small changes in c(m,[L]).

Allowing for k>0, the steady-state activity $A^{*}([L],m)$ becomes a complicated sigmoidal function of [L] and m [Eq. (A1)]. Intuition about the behavior of the sigmoidal dose response curve $A^{*}([L],m)$ can be gained by making a small modification to Eq. (3). If we multiply k by $\left(1+e^{\pm g(m)}\right)/2$, the methylation dependence of spreading and switching is identical, and the dependency of the steady-state activity on m and [L] combines neatly into the control parameter c(m,[L]). We then find

(5) $$A^{*}=\frac{k(c+1)+1-c-\sqrt{\left[k(c+1)+1-c{]}^{2}-4ck(1-c\right)}}{2(1-c)}.$$

When $m=m_{0s},A^{*}$ here is exactly equal to the steady state $A^{*}([L],m)$ predicted by the unmodified dynamics [Eq. (A1)], and when $m\neq m_{0s}$ they remain qualitatively similar and have nearly identical gain (Appendix D). This suggests any intuition gained from the modified dynamics should still apply to the unmodified model.

Plotting Eq. (5) as a function of c shows a sigmoid-like curve with inflection point at c=1 where there is precise symmetry between activity and inactivity [Fig. 1(c)]. As we reduce k, the curve becomes increasingly steep and switchlike, approaching the Heaviside function we found from our analysis as k→0. Carefully taking the k→0 limit of Eq. (5), we find that $\text{d}A^{*}/{\left.\text{d}c\right|}_{c=1}=(8k{)}^{-1}$; that is, for small k, the slope at the inflection point diverges like 1/k. This divergence is the source of large gain in our model.

This has exciting implications from an evolutionary design perspective. In order to maximize the gain of our sensor, all we need is separation of timescales between switching and spreading rates. Such a separation of timescales should be relatively straightforward to implement through evolution. In contrast, maximizing the gain in an Ising model requires that the coupling constant is tuned to a precise, finite critical value. The strength of that coupling, as well as its critical value, would likely be influenced by factors such as receptor packing density, inhomogeneities in the lattice, temperature, and other environmental factors, potentially challenging the effectiveness of evolutionary fine tuning. The LU model, by contrast, provides robust gain as long as spreading reactions are much faster than switching reactions.

Equations (4) and (5) also show how spreading, which is possible only between CSUs connected in an extended lattice, enhances the effects of methylation. Receptor sensitivity, and by extention the sensitivity of isolated CSUs, is described by the ligand concentration where p=1/2 or equivalently, where Δf([L],m)=0. Meanwhile, the sensitivity of connected CSUs is set by the ligand concentration where c=1, or equivalently, when g(m)-nΔf([L],m)=0. Thus, when g(m)≠0, the $k_{1/2}$ value of the kinase activity will be offset from that of the receptor.

We can confirm these predictions by fitting $A^{*}$ to experimentally measured dose response curves. Indeed, the mean-field LU model accurately captures the disparity between *in vitro* measurements of kinase activity and receptor binding dose response curves in reconstituted membrane vesicles [Fig. 1(d)], as well as between *in vivo* measurements of kinase activity in bacteria with and without the CheW-X2 mutation that prevents CSUs from combining into extended lattices [Fig. 1(e)]. In addition, the LU model is consistent with dose response curves from a complete range of methylation levels [6]. Differences in experimental procedures (strain differences with different subset of receptors expressed, temperatures of growth and measurement, in membrane vesicles vs live cells, etc.) mean that a single set of parameters cannot simultaneously fit data from all experiments. For convenience, we use parameters found from fitting the dose response curves in Ref. [6] throughout the paper (Table I). Details of the fitting procedure and parameters values for the other experiments can be found in Appendix A.

The equation for the steady-state kinase activity $A^{*}$ [Eq. (5)] and that for the full model [Eq. (A1)] is identical to the fraction of modified substrate in Goldbeter and Koshland’s model of zero-order ultrasensitivity, with the limit k→0 in our model corresponding to the case of enzyme saturation [27]. While mathematically equivalent to zero-order ultrasensitivity, LU has a different structural origin. In each case a forward reaction and a separate backward reaction work in opposition on the same substrate, and the system becomes ultrasensitive when the ratio of the forward to backward rate becomes relatively independent of the state of the substrate. For zero-order ultrasensitivity, this relative independence occurs when the competing reactions work at saturation. For the LU model, spreading of activity and inactivity both require a single active and inactive kinase to be neighbors on the lattice, so the ratio of their rates is independent of the average activity. The ratio of spontaneous activation and deactivation rates does depend on the average activity, but these processes are much slower than spreading and are analogous to a slight deviation from precisely balanced saturated enzymatic reactions in zero-order ultrasensitivity.

### C. k controls key features of response dynamics

Analysis of the mean-field model is useful for providing qualitative insights, but because it ignores spatial correlations, its quantitative predictive power is limited. We turn to simulations to understand how responses depend on parameters.

**FIG. 2.**
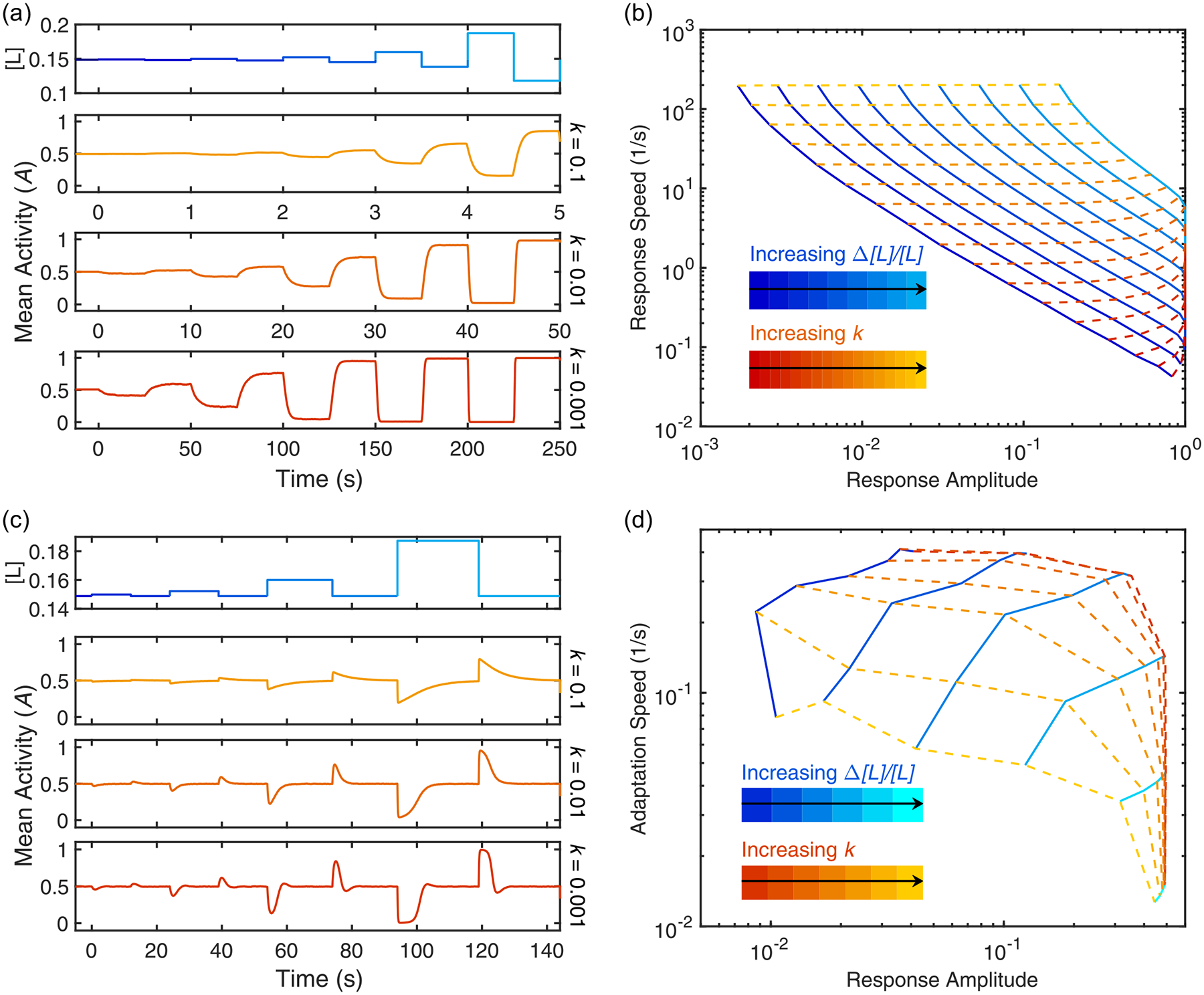
Response and Adaptation Dynamics. (a) Averages of 1000 simulated response to step changes in ligand concentration at fixed methylation show how responses become larger and slower as k decreases. Note that the x axis is scaled differently for each value of k. The ligand profile for each simulation was identical, except that the durations of the steps were adjusted to reflect the varying response rates for different k. (b) Plot of response amplitude vs speed at fixed methylation. Response amplitude was calculated as the difference between $A^{*}$ at $L=L_{1/2}\left(1+\Delta L/L_{1/2}\right)$ and $L_{1/2}/\left(1+\Delta L/L_{1/2}\right)$, where $L_{1/2}=0.15\;\text{mM}$ is the ligand concentration corresponding to $A^{*}=1/2$. Response speed was found by fitting the activity time series following such a change in ligand concentration to an exponential function $f\left(t\right)=A+B\;\text{exp}\left(-t/\tau_{r}\right)$. (c) Similar to (a), but adaptation is allowed, so methylation is no longer fixed. (d) Plot of response amplitude vs adaptation time. Adaptation speed is $1/\tau_{a}$, where $\tau_{a}$ is the time at which A(t)-A(0)=max[A(t)-A(0)]/e and t is the time since the stimulus. Response amplitude is given by max[A(t)-A(0)]. For parameter values see Table I.

We simulated the activity response of the LU model to step stimuli, both with and without adaptation, for different values of k [Figs. 2(a) and 2(b)]. In the nonadapting case, the magnitude of the response for a given stimulus is taken to be the change in activity when we take c→1/c. We find the speed of the response by fitting the activity to an exponential. In keeping with our findings from mean field, the magnitude of response is roughly proportional to 1/k, at least until the activity response begins to saturate. The response speed is roughly proportional to k, indicating a tradeoff between response speed and amplification. Lowering k to increase gain also slows down the response. Unfortunately, measurements of the response time in cells via FRET are poorly constrained and limited by the dynamics of the CheY and CheZ FRET pair, which may be significantly slower than the dynamics of the lattice.

In contrast, no such tradeoff seems to exist when it comes to adaptation. The speed of adaptation increases as the system becomes more sensitive to stimuli. While the adaptation dynamics have no explicit dependence on k, they do depend on kinase activity. The higher the methylation rate the further the kinase activity is from its steady-state value. When gain increases, the same change in ligand will create a larger shift in activity, so that the methylation rate will be higher. However, the steady-state methylation level depends only on ligand concentration, so changing k doesn’t change the amount of methylation required to reach a new steady state. Thus, adaptation is faster at larger amplification and smaller k.

### D. Proximity to a critical point amplifies fluctuations and causes two-state switching

**FIG. 3.**
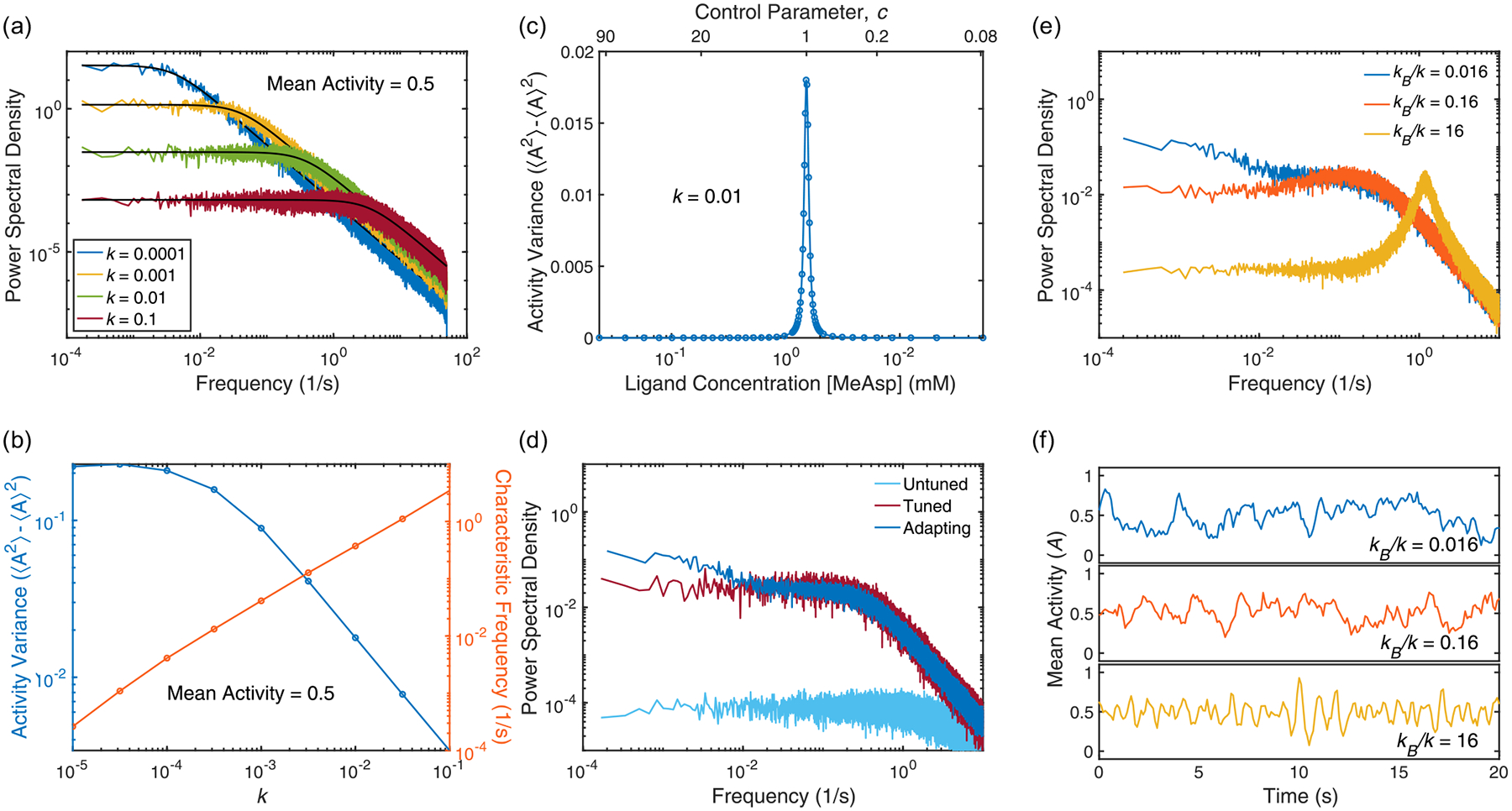
Large fluctuations from the LU model. (a) Power spectral density of simulated kinase activity fluctuations at constant ligand concentration and methylation, for increasing values of k. Black lines show fits to a Lorentzian distribution. (b) The characteristic frequency of fluctuations in nonadapting cells is linearly proportional to k. The variance shows power law scaling with k when k is large. When k becomes sufficiently small, the variance saturates due to the onset of two-state switching. (c) For nonadapting cells, variance is sharply peaked near c=1 and small elsewhere. (d) Power spectra show very small fluctuations in nonadapting cells without tuned ligand concentrations (mean activity = 0.1). When cells have a control parameter close to 1 (mean activity = 0.5), either through adaptation or tuning of ligand concentration, fluctuations are much larger. (e) Three different regimes of adapting cells. $k_{R}/k_{B}$ is held constant throughout. When adaptation is much slower than k, two distinct timescales are visible in the power spectral density. When adaptation is faster than k, the PSD is peaked, indicating the presence of oscillation. Between these two limits, the PSD appears Lorentzian with a single timescale proportional to k. (f) Example time series of cells corresponding to the three regimes depicted in E. For parameter values see Table I. All mappings from [MeAsp] to c assume $m=m_{0s}$.

The LU model generates large activity fluctuations in proximity to its critical point, which occurs at k=0 and c=1. We simulated 100 000 s of activity in cells with different values of k and fit the power spectra of their fluctuations to a Lorentz distribution [Fig. 3(a)]. We held methylation and ligand levels constant, so fluctuations were due only to stochastic kinase dynamics as would be the case in ΔcheRΔcheB cells. As we reduced k the characteristic timescale and variance of the fluctuations both increased, showing power-law scaling consistent with behavior near a critical point at k=0 [Fig. 3(b)]. For small k, the size of the fluctuations is comparable to that of the entire system, consistent with single cell measurements [7,8]. The saturating variance seen for very small k is because of this—the finite lattice size means that once fluctuations encompass the entire system, they cannot grow any larger.

In agreement with experimentally observed fluctuations [7,8,20], the LU model produces large fluctuations only when activity is brought to an intermediate level through adaptation or fine-tuning of ligand concentration. Indeed, the variance of the fluctuations is sharply peaked in the vicinity of c=1 [Fig. 3(c)]. By bringing c close to 1, adaptation thus leads to large fluctuations. To do this without adaption requires fine-tuning the ligand concentration. This behavior is reflected in the power spectra. Adapting cells and nonadapting cells with finely tuned ligand concentrations have much larger fluctuations than nonadapting cells without fine tuning [Fig. 3(d)].

Adaptation dynamics also introduce noise via stochastic methylation, leading to small fluctuations in c that change the shape of the power spectrum [Fig. 3(e)]. When the sum of methylation rates, $k_{B}+k_{R}$, is much less than k, the system amplifies these fluctuations in c, adding a second, slower timescale to the power spectrum near the methylation frequency. When the sum of methylation rates is similar to k, the two timescales converge and the power spectrum again appears Lorentzian. If methylation rates increase further, adaptation is fast enough to respond to individual, stochastic changes in activity, suppressing low-frequency fluctuations. However, it also allows for feedback between methylation and activity that leads to a high-frequency peak in power spectrum and visible oscillations in the time series data [Fig. 3(f)]. These oscillations are related to the oscillations predicted in Ref. [34]. However, because their model assumes the kinase dynamics are infinitely fast compared to methylation dynamics, their system only displays oscillations below the critical point when hysteresis provides a second timescale. Experiments might be able to observe these oscillations by overexpressing adaptation enzymes in cells.

Two-state switching can occur in the LU model whenever fluctuations are large enough to push the system into a state where all the kinases are either active or inactive. Once the activity is uniform across the lattice, spreading can no longer occur. The lattice can leave this uniform state only via a spontaneous switching reaction. Because switching is much slower than spreading, once the lattice becomes uniformly active or inactive it will stay in that state for a relatively long time. This creates the appearance of two-state switching similar to what has been observed experimentally [7,21].

**FIG. 4.**
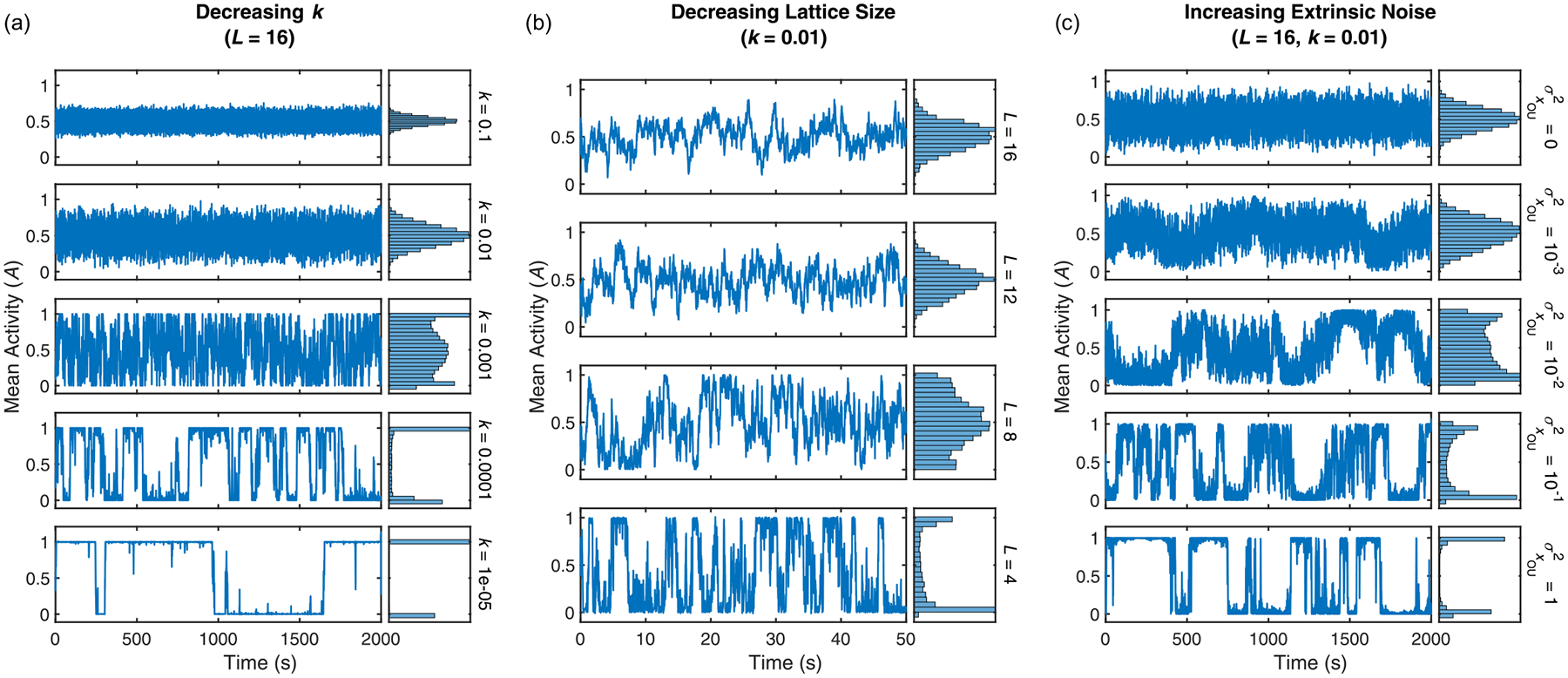
Emergence of two-state switching. (a) Simulated time series with mean activity set to 0.5 and decreasing k from top to bottom. As k decreases, fluctuation size and timescales grow. Once fluctuations are large enough to reach the boundaries of the system (A=0 or A=1), switching begins to occur. (b) As in (a), but now decreasing the linear dimension of the lattice, L, from top to bottom. As L decreases, the size of fluctuations relative to the total number of kinases increases. Switching occurs when the system is small enough that the fluctuations reach the boundaries. The x axis is zoomed in to make high frequency switching clear. (c) As in (a), but we have introduced noise into the control parameter. Variance of the noise increases from top to bottom. Sufficiently large noise in the control parameter induces two-state switching in what would otherwise be a nonswitching system. Here k=0.01, equivalent to the second plot in (a). For parameter values see Table I.

Two simulation parameters control the relative size of fluctuations, and thus the presence of switching. Reducing k increases the size of fluctuations, eventually leading to two-state switching [Fig. 4(a)]. Decreasing the lattice size can also drive switching [Fig. 4(b)]. The values of k which produce two-state switching are significantly smaller than those found earlier from fitting dose response curves. However, the dose response curves fitted earlier all come from population average measurements. If these cells have variation in their sensitivity to ligand, the averaged dose response curves will appear to have smaller gain than the average gain of individual cells. This effectively leads to a larger fitted k. Furthermore, individual cells can deviate substantially in their observed amplification [7,9]. Given that only about 15% of cells exhibit two-state switching even in carefully tuned ligand concentrations [7], we should not be surprised that parameters which produce two-state switching differ from those of population averaged measurements.

While internal dynamics are sufficient to produce fluctuations and two-state switching similar to experiments, sources of noise external to the LU model may also contribute to these behaviors. Indeed, the addition of noise to the transition rates in systems with zero-order ultrasensitivity has been shown to produce repeated transitions between bistable states [35]. To investigate this possibility we introduced external noise by multiplying the spreading rate of activity by $e^{x_{\text{OU}}}$, where $x_{\text{OU}}$ is a variable obeying Ornstein-Uhlenbeck dynamics with zero mean, a defined variance, and a characteristic timescale. Increasing the variance of $x_{\text{OU}}$ made fluctuations larger, eventually producing two-state switching in simulations that would not otherwise have it [Fig. 4(c)]. Many known processes might contribute to external noise, such as the lattice exchanging receptors and kinases with its surrounding or performing dynamic structural rearrangements [36]. Additionally, recall that only a small fraction of cells exhibit two-state switching [7]. If any of the sources of this heterogeneity are themselves dynamic, they could also contribute to external noise.

## III. SUMMARY AND DISCUSSION

We have introduced a lattice model for the large gain observed in *E. coli* chemosensing. The LU model uses nonequilibrium reactions between CSUs to explain the large gain and adaptation seen in wild-type cells’ kinase activity. Our model can also account for the reduced gain and adaptation seen in lattice mutants and in receptor binding assays, and it recapitulates the large fluctuations and two-state-switching seen in single-cell experiments. Finally, unlike other models where gain arises from proximity to the critical point of a lattice model, the LU model does not require any fine-tuned allosteric interactions between neighboring receptor or kinases, achieving large gain as long as a switching reaction is much slower than a spreading reaction.

The critical point in our model reproduces the most salient features of fluctuations measured in single cells: large slow fluctuations and two-state switching in a subset of nonadapting cells [7,8,21]. The magnitude of these fluctuations is larger than expected in MWC-like models where kinases act independently or in small groups. Indeed, the fluctuations and two-state switching are difficult to reconcile with any model without a critical point. Both our model and Ising models contain such a critical point. However, the LU model achieves that critical point without fine-tuning coupling parameters, while the Ising model requires its coupling constant to be close to a specific finite value.

Other biological sensory systems also exploit the divergent susceptibility found near a critical or bifurcation point to amplify weak signals. Hair cells amplify faint sounds in the inner ear by tuning it close to a Hopf bifurcation [37–41]. We have argued that pit vipers use a saddle-node bifurcation to amplify milli-Kelvin changes in the temperature of their sensory organ into a robust neural response [42], and that olfactory receptor neurons in fruit flies exploit a bifurcation to extract odor timing information for navigation [43]. In all three cases, it has been argued that order-parameter-based feedback, originally introduced in the context of self-organized criticality [44,45] and bistability [46,47], allows the system to maintain proximity to the critical or bifurcation point without the need for fine-tuning. In our model, this feedback from the order parameter back onto the control parameter is implemented by the activity dependence of methylation dynamics [32,48,49].

**FIG. 5.**
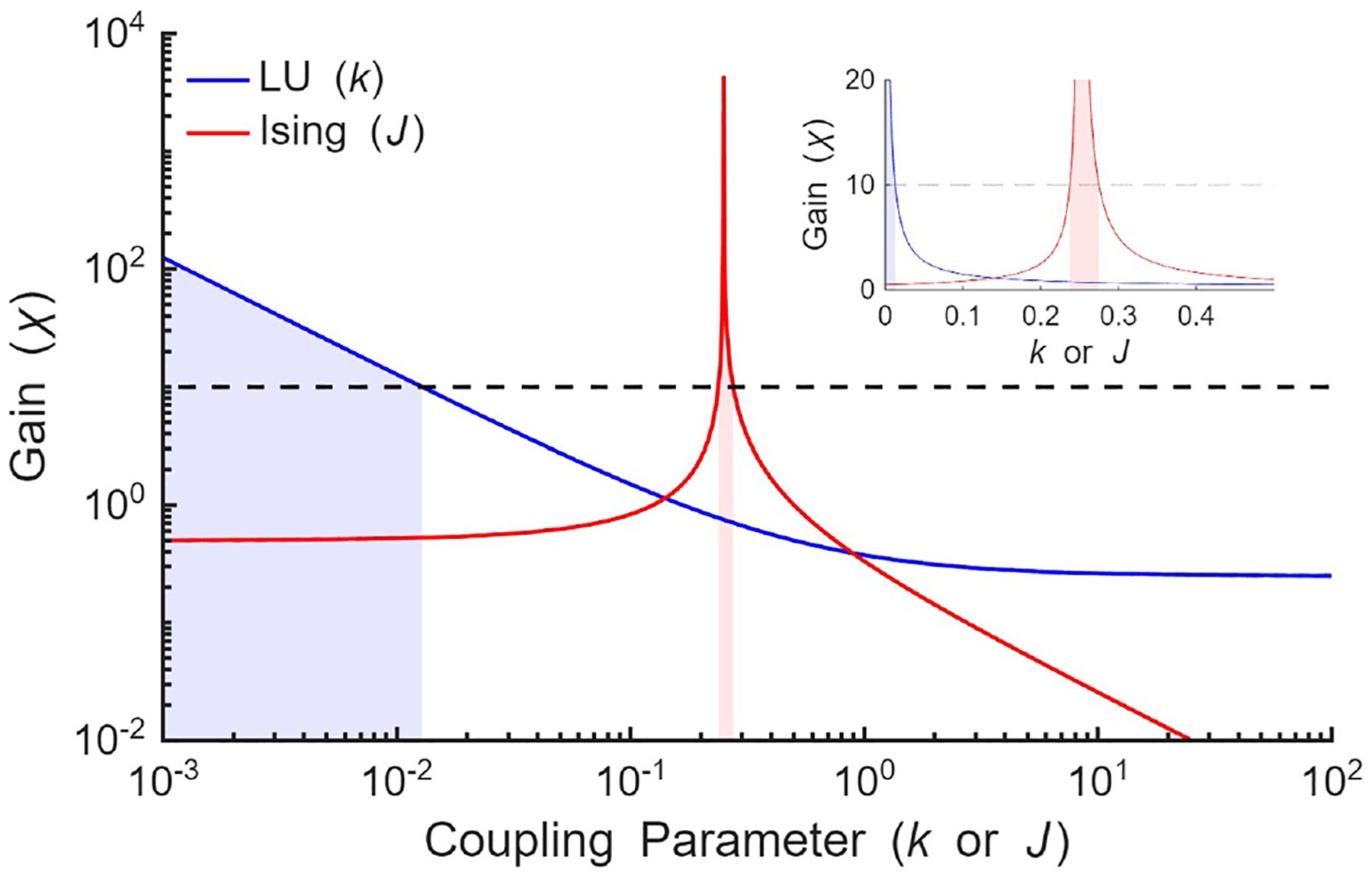
LU model can achieve high gain without finely tuned interactions. Both models have a codimension 2 critical point, requiring two parameters to be near a critical value to achieve large gain through a diverging susceptibility, χ. In both Ising and LU models of the chemosensory array, perfect adaptation through methylation/demethylation of receptors tunes one of these parameters (c=1 in the LU model, h=0 in the Ising model). We plot the mean-field values for χ as a function of the second parameter (k for the LU model in blue, J for the Ising model in red). The calculations are shown in Appendix E. In the Ising model, large gain arises only when J is near to a critical value, $J_{c}$. By contrast, because the LU model’s critical point is at k=0, large gain requires only that k≪1. The region of parameter space where gain is large (represented here by χ>10) are shaded. For the LU model, though, the region is very large in log space. It can be reached through simple timescale separation because it needs only k≪1. Within this region, large gain is robust to significant relative changes in k.

Barkai and Leibler showed in 1997 that precise adaptation in bacterial chemotaxis is a result of network connectivity. Thus, it does not require parameter fine-tuning and is robust to parameter variation [48]. Another key feature of chemotaxis signaling is large gain. In Ising models, large gain arises from the fine-tuning of an allosteric interaction parameter. In our LU model, large gain occurs whenever spreading dominates over switching. This can be achieved through a timescale separation by having the spreading chemical reaction occur much faster than the switching one. This timescale separation is robust to far larger variation than the fine-tuning of allosteric interactions needed in the Ising model (Fig. 5). This is distinct from the timescale separation between response and adaptation via methylation, which is shared by all models including ours.

That coupling between CSUs in the LU model is explicitly out of equilibrium distinguishes it from other models. The reduced gain and adaptation measured in receptor binding related to kinase activity [23,24] is captured by the LU model [Fig. 1(d)] but cannot be explained by equilibrium models [25]. Other models have also explained this by introducing nonequilibrium interactions within CSUs [22,25]. However, kinase activity in certain CheW mutants also show reduced adaptation and gain [26], suggesting that by disrupting connections between CSUs these mutants also disrupt the nonequilibrium process responsible for enhancing gain and adaptation. This implies that the nonequilibrium process involves the coupling between neighboring CSUs as in the LU model, not just interactions within individual CSUs.

The mechanism responsible for nonequilibrium coupling (i.e., spreading) between CSUs is quite different from the allosteric interactions assumed to explain previous equilibrium couplings. One possible mechanism involves P1, the phospho-accepting domain of CheA. P1 is connected to the rest of the CheA dimer by an extended flexible linker. This linker allows P1 to be phosphorylated by the P4 domain of the opposite CheA in the dimer pair, and it is long enough that it might facilitate reactions with neighboring core units as well [50]. Thus, spreading could occur via direct phosphotransfer reactions between neighboring CheA mediated by P1.

Our model here deals only with homogeneous receptor types, but it could easily be expanded to explore the implications of mixed receptor types. Modeling MWC clusters with mixed receptor types has been done [2], so it would be relatively straightforward to incorporate that into the receptor clusters in the LU model. Because the number of receptors within each CSU is relatively small, we expect that heterogeneity between receptor composition of different CSUs would still allow for different receptor types to adapt independently to different ligands.

The molecular implementation of spreading is poorly constrained due to the large number of states and interactions described among the proteins in the signaling lattice. However, future experiments may be able to improve the situation. For instance, P1-mediated interaction could be tested by shortening the flexible linkers, making interactions between neighboring kinases less likely. This could reduce gain, but might entirely stop signal transduction first. As for lengthening linkers, experiments have already shown that mutants whose P1 and P2 domain are entirely separate from the lattice and soluble in the cytoplasm are still able to chemotax [51], but quantitative probes of signaling activity in these cells have not been performed. If the reduced gain and adaptation seen in CheW mutants [26,52] is because core units are too spatially separated for their linkers to reach each other, perhaps mutants with soluble P1-P2 domains can partially rescue large gain in the CheW mutants. Intriguingly *Vibrio cholerae* has far fewer and therefore sparser CheA [53], but their linker is significantly longer [54–56], potentially allowing CheAs to interact over longer distances.

Other experiments may be able to probe different features of the LU model. The LU model predicts that balanced overexpression of adaptation enzymes CheR and CheB should lead to oscillations in kinase activity [Figs. 3(e) and 3(f)], although this prediction may not be unique to the LU model. Additionally, the nonequilibrium nature of the proposed spreading interaction means that experiments restricting the free energy available via ATP hydrolysis should meaningfully affect signaling. Further theoretical work is needed to clarify precisely how limiting available energy would impact measurable signaling characteristics such as response time, gain, and variance.

The LU model presents a qualitatively different mechanism for achieving sensory gain through proximity to a critical point. LU dynamics are driven by directed chemical reactions between neighboring signaling enzymes rather than by thermal fluctuations. The result is a mechanism for amplification that resembles zero-order ultrasensitivity. This mechanism seems to be more robust—acting as an effective amplifier without the finely tuned energetic coupling that is required for analogous equilibrium critical points. While coarse-grained, the LU model provides insights for interpreting the structure, function, and design principles of receptors and kinases in the chemosensory array.

## Figures and Tables

**FIG. 6.**
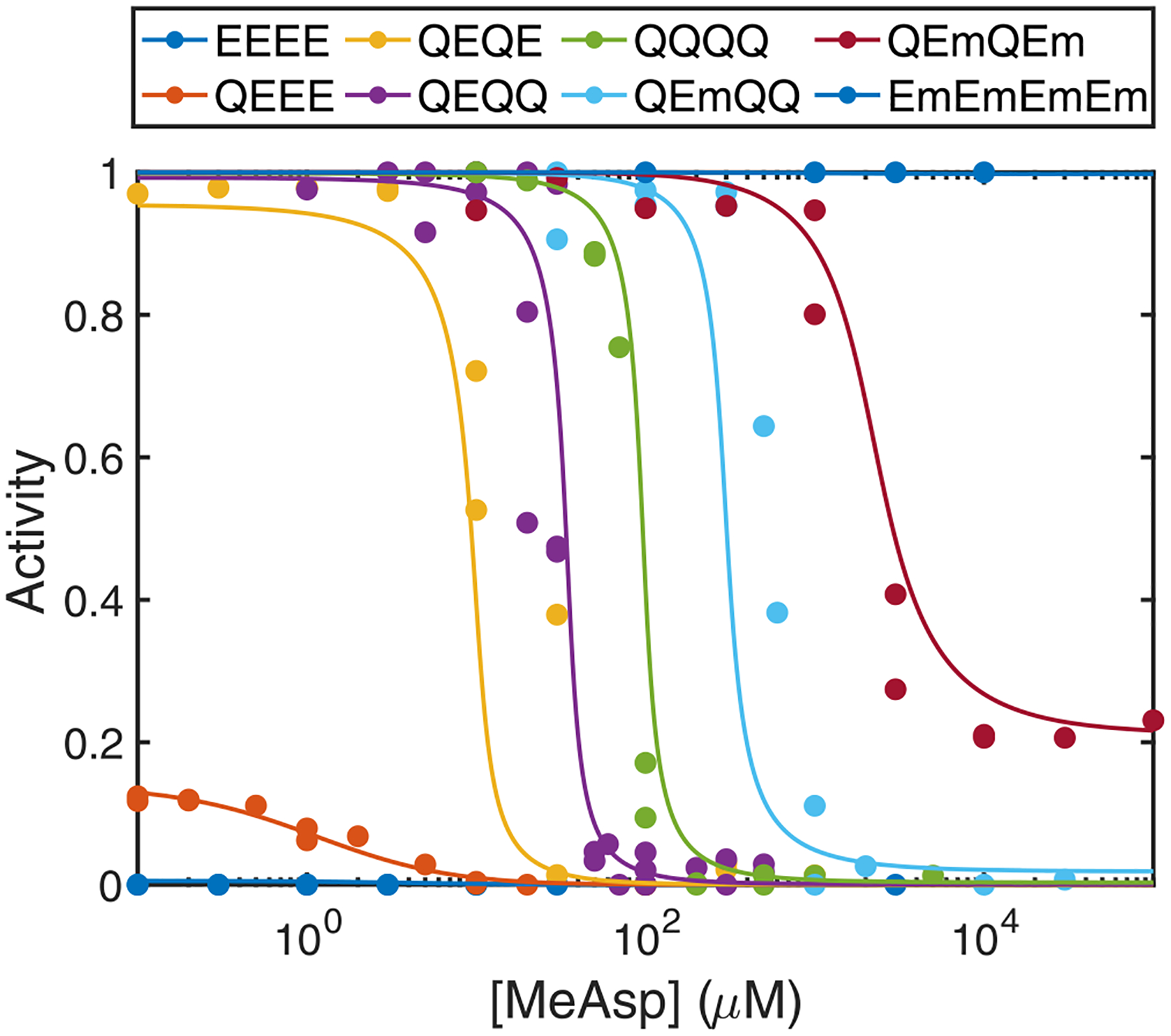
Fits to experimental dose response curves from Ref. [6].

**TABLE I.** Simulation parameters. These values were used in all simulations unless otherwise specified. They were found by fitting data from [6]. See Appendix A for details.

Name	Value	Description
v	100 s^−1^	Base Spreading Rate
$k_{R}$	0.024 s^−1^	Methylation Rate
$k_{B}$	0.01 s^−1^	Demethylation Rate
k	0.1	Ratio of Switching to Spreading
n	2	Receptor MWC Factor
$m_{0r}$	−0.28	Receptor Crossover Methylation Level
$\alpha_{r}$	0.99 kT	Receptor Free Energy per Methylation
$m_{0s}$	2.21	Spreading Crossover Methylation Level
$\alpha_{s}$	0.92	Spreading Effect per Methylation
$K_{a}$	474μM	Dissociation Const. of Activating Receptor
$K_{i}$	10μM	Dissociation Const. of Inactivating Receptor

**FIG. 7.**
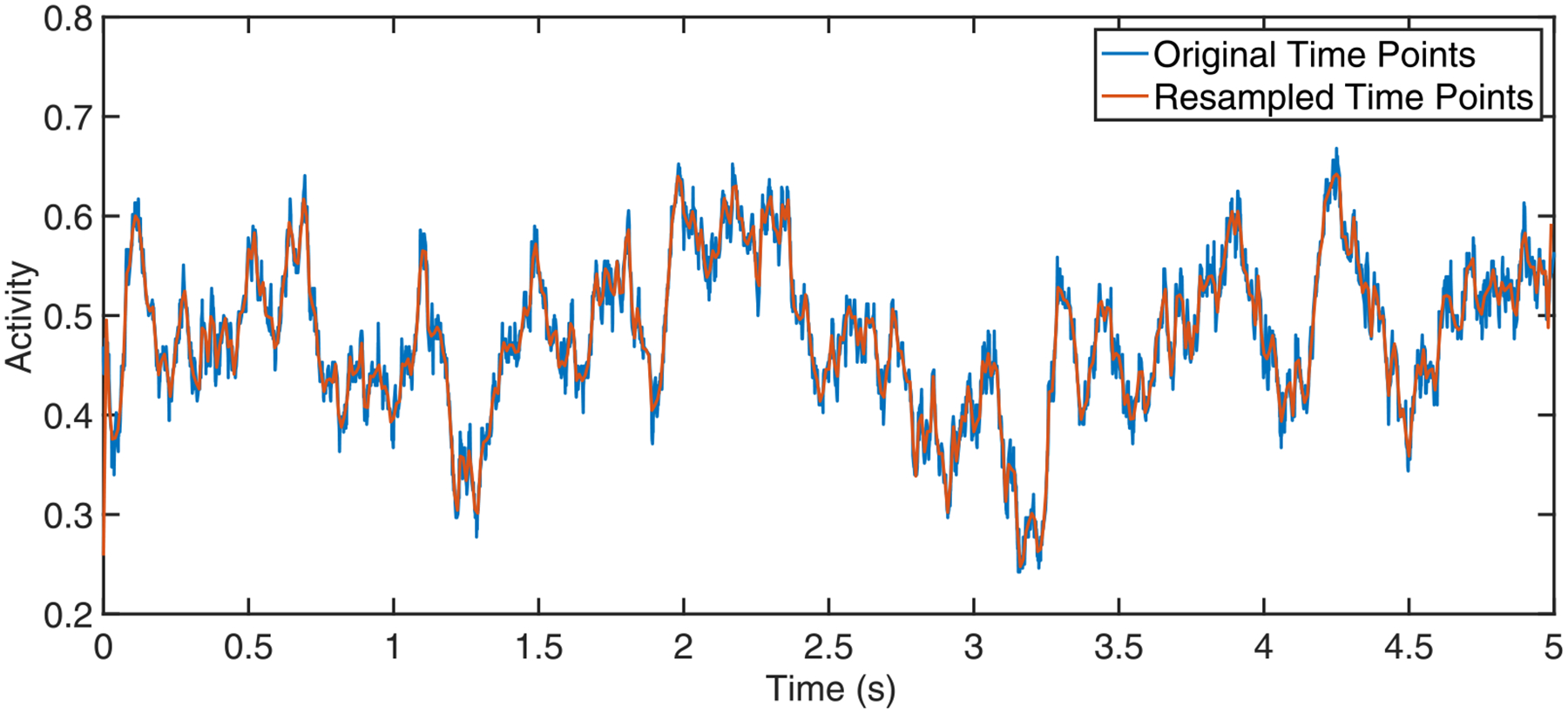
Comparison of original and resampled data. Results are identical, except on timescale less than 0.1 s, which are not probeable using current FRET techniques.

**FIG. 8.**
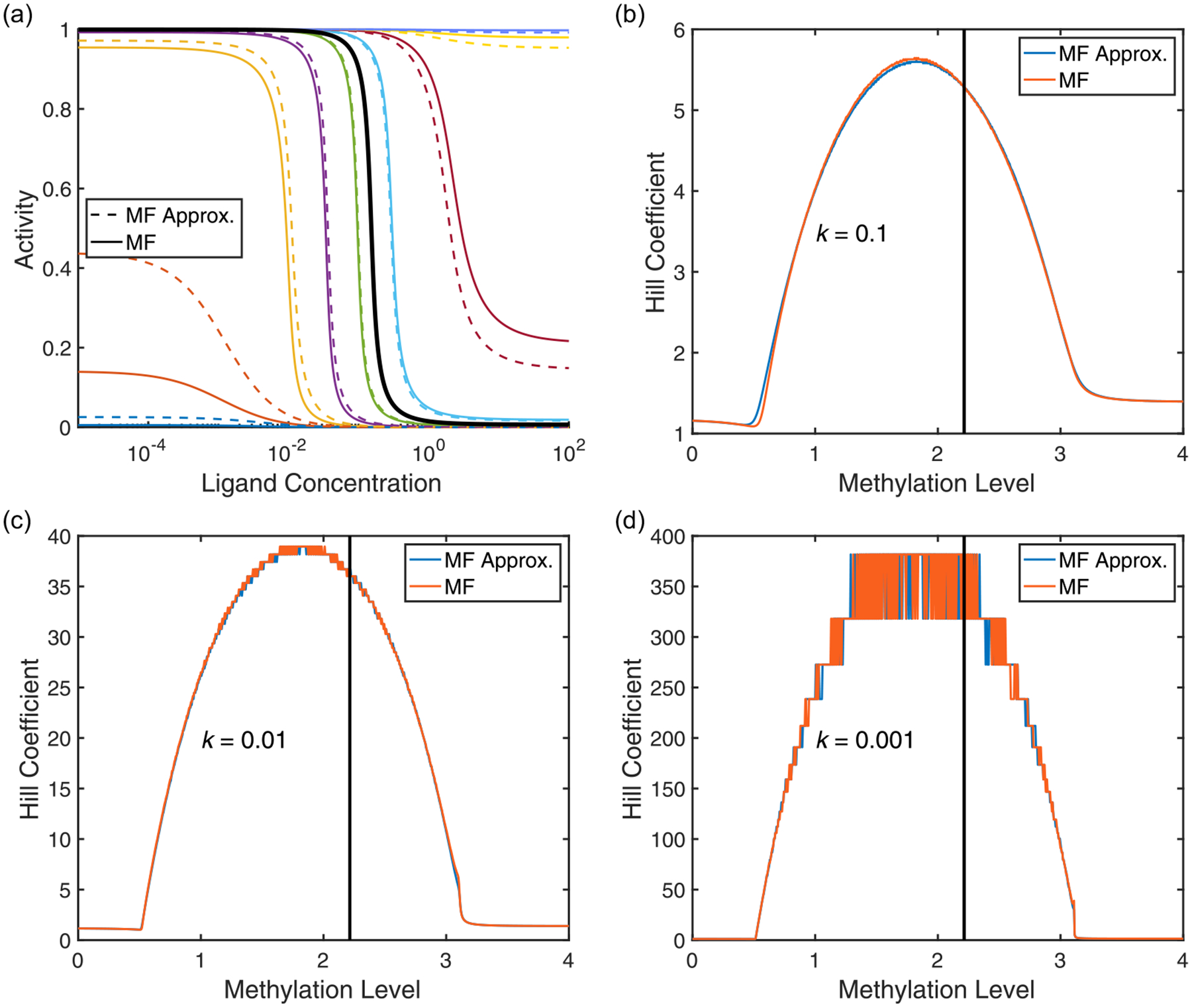
Comparing predictions of exact and approximate equations for the mean-field steady-state activity. (a) Dose response curves for exact and approximate solutions show reasonable good agreement, diverging significantly only for m far from $m_{0s}$. Methylation levels for dose response curves start at m=0 in blue at the bottom left, with m increasing by 0.5 for each new color until it reaches purple in the top right at m=4. The thick black curve corresponds to $m=m_{0s}$ (b)–(d) Hill coefficients as a function of m for different values of k. The vertical black lines show the location $m=m_{0s}$. Agreement between the approximate and exact solution is again very good, even when m is far from $m_{0s}$. Discrete step sizes seen at large Hill coefficients is an artifact of ligand step size used in generating dose response curves.

## Data Availability

The data that support the findings of this article are openly available [57].

## APPENDIX A: DETERMINING PARAMETER VALUES

### 1. Fitting dose response curves

For each data set that we fit, we used the same general procedure. Each dose response curve in the data set was normalized by its mean value, and data corresponding to specific methylation mutants were assigned a specific value of m for fitting (EEEE: m=0, QEEE: m=0.5, QEQE: m=1, QQQE: m=1.5, QQQQ: m=2, QEmQQ: m=2.5, QEmQEm: m=3, EmEmEmEm: m=4). All curves in a given data set were then simultaneously fit to the mean-field equation for the LU model using MATLAB’s nonlinear least-squares curve-fitting function, see Fig. 6.

Except for CheW mutants, all kinase activity dose response curves were fit to the steady-state activity for the full model

(A1)
$$A^{*}=\frac{1-c+\frac{2k}{1+e^{g(m)}}+\frac{2ck}{1+e^{-g(m)}}-\sqrt{{\left(1-c+\frac{2k}{1+e^{g(m)}}+\frac{2ck}{1+e^{-g(m)}}\right)}^{2}-4(1-c)\left(\frac{2ck}{1+e^{-g(m)}}\right)}}{2(1-c)},$$

with all terms defined in the main text. Assuming that the rate of spreading in CheW mutants is zero, resolving the dynamical equations reveals that the steady-state activity of these mutants is just

(A2)
$$A_{\text{CheW}}^{*}=p([L],m).$$

Based on the differing ligand affinities of the activating and inactivating receptors, the equation we fit to the ligand binding curves was

(A3)
$$b=\frac{L}{K_{i}+L}[1-p([L],m)]+\frac{L}{K_{a}+L}(p).$$

Parameters found from fitting receptor binding curves [24], CheW mutant activity [26], and cells with WT CheA and CheW [6] are different, see Table II. However, these are because of differences in the experimental systems, leading to corresponding differences in dose response curves which necessitate difference in fit parameters. Reference [24] measured receptor binding and kinase activity from membrane vesicles with reconstituted signaling arrays. Because this environment is so different from that of live cells, it’s not surprising that their dose response curves would also differ. There are also important differences between the strains. Reference [6] used cells deleted for Tsr and Tap, whereas Ref. [26] used cells deleted for all five chemotaxis receptors. While the expression of Aer and Trg are small compared to Tsr and Tar [58], they can still meaningfully shift dose-response curves. Additionally the expression of methylation-locked Tar was from a plasmid in Ref. [26], while in Ref. [6] expression was from the native loci on the chromosome. These differences are known to impact the signaling properties such as EC_50_ of kinase activity. Finally, dose response measurements in Ref. [6] were made at 22 °C, while they were made at 30°C in Ref. [26]. Such temperature differences are known to significantly impact activity dose response curves in *E. coli* [59].

### 2. Finding the remaining parameters

After fitting the dose response curves, we still need to determine the base spreading rate ν and the methylation and demethylation rates $k_{R}$ and $k_{B}$. The steady-state activity level after adaptation, $\overset{‾}{A}=2/\left(1+\sqrt{1+4k_{B}/k_{R}}\right)$, determines the ratio $k_{R}/k_{B}$. We chose $\overset{‾}{A}=0.3$ in rough agreement with the average of cells from Ref. [60], corresponding to $k_{R}/k_{B}=2.4$. The total of the (de)methylation rates, $k_{R}+k_{B}$, determines the adaptation timescale, which is roughly 10 s [60]. After simulating the response of adapting cells to a 10% change in ligand concentration for a variety of values, we found that $k_{R}+k_{B}=0.034{\text{s}}^{-1}$ roughly corresponds to an adaptation timescale of 10 s. Because of significant variability between individual cells, a more precise fitting procedure for the (de)methylation rates would provide meaningfully better information.

**TABLE II.** Parameters found from fitting data from Refs. [24] and [26].

Parameter	Description	Values for *in vitro* receptor binding [24]	Values for *in vivo* CheW mutants [26]
k	Ratio of Switching to Spreading	0.1	0.1
n	Receptor MWC Factor	2	2
$m_{0r}$	Receptor Crossover Methylation Level	−3.35	0.69
$\alpha_{r}$	Receptor Free Energy per Methylation	0.34 kT	2.09 kT
$m_{0s}$	Spreading Crossover Methylation Level	1.72	−0.33
$\alpha_{s}$	Spreading Effect per Methylation	1.57	0.56
$K_{a}$	Dissociation Const. of Activating Receptor	4.5μM	126 mM
$K_{i}$	Dissociation Const. of Inactivating Receptor	0.85μM	9μM

Because the response timescale of kinase activity is smaller than the time resolution available from FRET microscopy [60], it cannot be effective constrained by FRET measurements. This, in turn, means that the base spreading rate v is also poorly constrained, with the only requirement being that our system responds faster than the resolution of FRET microscopy, roughly 500 ms. We chose v=100Hz, comfortably above this minimum rate. These additional parameters are summarized in Table III.

## APPENDIX B: DESCRIPTION OF SIMULATIONS

### 1. Setup and initialization

In our simulation, core signaling complexes (CSCs) are located on the sites of a square lattice. There are two values associated with each CSC on the lattice: kinase activity (A) and methylation level (m). The kinase activity takes a value of 0 or 1, corresponding to the inactive or active state, respectively. The methylation level takes a value between 0 and 4. Since there are 12 receptors (two trimers of dimers) in each CSC, we discretize methylation levels into steps of dm=1/12, corresponding to the methylation of a single site of one receptor. A methylation level of 0 or 4 means that every receptor in the CSC is either fully demethylated or methylated. There is also a value p associated with the state of the receptor in each CSC. We assume that the dynamics of the receptor state are in fast equilibrium relative to the other dynamics of the system, so rather than take a value of 0 or 1 corresponding to the inactivating or activating states, it takes the average state, p, somewhere between 0 and 1:

(B1)
$$p([L],m)={\left(1+e^{n\Delta f([L])}\right)}^{-1}.$$

(B2)
$$\Delta f([L],m)=\Delta f_{0}(m)+\text{log}\left(\frac{1+[L]/K_{i}}{1+[L]/K_{a}}\right).$$

To initialize the simulation with adaptation, the kinase activity and methylation level of each CSC is determined randomly. The receptor state is determined based on the initial ligand concentration. If we are simulating an nonadapting mutant (e.g., QEQE), initialization of the kinase activity and the receptor state occurs as above, but the methylation level of each CSC is set to a constant value that is determined by the mutant in question.

**TABLE III.** Additional parameter values chosen for use in our model.

Parameter	Description	Value
v	Base Spreading Rate	100 s^−1^
$k_{R}$	Methylation Rate	0.024 s^−1^
$k_{B}$	Demethylation Rate	0.01 s^−1^

### 2. Dynamics

Dynamics are then simulated using a Gillespie algorithm [33] with unique reactions for each CSC. The propensity for reactions at the ith CSC is given by the following: an inactive kinase becoming active,

(B3)
$$r_{0\to 1}^{A_{i}}=p\left(k+\frac{1+e^{g\left(m_{i}\right)}}{4}\sum_{j\in n.n.}A_{j}\right),$$

where m is the methylation level of the CSC, other variables are defined as in the main paper, and the sum is over the activity values of nearest neighbor kinases. The propensity for active kinases to become inactive is given by

(B4)
$$r_{1\to 0}^{A_{i}}=(1-p)\left[k+\left(1+e^{-g\left(m_{i}\right)}\right)\left(1-\frac{1}{4}\sum_{j\in n.n.}A_{j}\right)\right].$$

The propensity to increase the methylation by dm is given by

(B5)
$$r_{+dm}^{m_{i}}=k_{R}\left(1-A_{i}\right).$$

The propensity to decrease the methylation by dm is given by

(B6)
$$r_{-dm}^{m_{i}}=k_{B}A_{i}\frac{1}{N}\sum_{j=1}^{N}A_{j}.$$

Here the sum is over every lattice site, making the demethylation rate proportional to both the single site activity and the average over the entire lattice. This accounts for the activation of CheB via phosphorylation by CheA.

Sometimes, rather than simulate the lattice at a specific methylation level and/or ligand concentration, it is convenient to just set the control parameter $c=e^{m_{0}+\alpha m_{i}}\;p/(1-p)$, which combines the influence of methylation and ligand into a single parameter. In this case the propensity for methylation and demethylation are unchanged, while those of activation and inactivation of kinases becomes

(B7)
$$r_{0\to 1}^{A_{i}}=\frac{c+1}{2}\left(k+\frac{1}{4}\sum_{j\in n.n.}A_{j}\right)$$

and

(B8)
$$r_{1\to 0}^{A_{i}}=\frac{\frac{1}{c}+1}{2}\left(k+1-\frac{1}{4}\sum_{j\in n.n.}A_{j}\right).$$

The prefactors were chosen to correspond with the case where p=0.5. Because the total rates depend slightly on p when c≠1, we need to choose a specific value of p when running these simulations.

In a traditional Gillespie algorithm, all of these individual propensities are calculated at the beginning of each step of the simulation. Then a single reaction is chosen to take place, with the probability for a given reaction to be chosen is proportional to its propensity. The amount of time that passes between each reaction would be chosen from an exponential distribution whose timescale is equal to the inverse of the sum of all of the reaction propensities.

To speed up simulation times, a form of tau leaping was implemented. Rather than recalculate all the propensities after each reaction, we perform multiple reactions drawing from the same propensity function. The number of reactions performed was chosen to be

(B9)
$$n=0.1\;\text{min}\left(\sum_{j}A_{j},N-\sum_{j}A_{j}\right),$$

rounded up. This guarantees that the total number of active or inactive kinases will not change by more than 10%, ensuring that our approximation of a constant propensity function is good for all the reactions. The time step is then corresponding increased by a factor of n.

The units in our simulation are defined so that the spreading rate of activity at c=1 is equal to 1. Thus, one unit of time in the simulation is equal to 1/s s, where s is the spreading rate in units of inverse seconds.

Before taking data, if adaptation is occurring, the model is run for $3/\left(k_{R}+k_{B}\right)$ s in order to equilibrate. If adaptation is not occurring (i.e., $k_{R}=k_{B}=0$), the simulation is run for 100 s to equilibrate.

Once the system has equilibrated, we run through whatever experimental protocol we require. A protocol is defined as a series of ligand concentrations and a length of time the system experiences each ligand concentration in that series. The ligand concentration chosen for equilibration should be the same as the first ligand concentration in the series. The system is run for the specified amount of time at that concentration, then changed to the next one, until the protocol is complete.

### 3. Resampling

At this point the model has output a time series of methylation levels and kinase activities, but the spacing between timepoints is nonuniform. For ease of analysis, we then resample the data at 100 Hz using MATLAB’s resample function to create a uniformly sampled time series (Fig. 7). From there, it is easy to calculate the power spectrum, or average over many different realizations of the same protocol.

### 4. Implementing external noise

To implement sources of noise in the control parameter that are external to our model, we multiplied $r_{0\to 1}^{A_{i}}$ by an additional term, $e^{x_{\text{OU}}}.x_{\text{OU}}$ is described by an Ornstein-Uhlenbeck process with a relaxation time τ and a noise scale D. Its value is updated at every time step of the simulation obeying the following rule:

(B10)
$$x_{\text{OU}}\left(t+dt\right)=x_{\text{OU}}\left(t\right)\left(1-\frac{dt}{\tau }\right)+D\text{N}\left(0,1\right)\sqrt{dt}.$$

Here N(0,1) denotes a random variable drawn from a normal distribution with mean 0 and variance 1.

## APPENDIX C: DEPENDENCE OF RESPONSE TIMES ON RECEPTOR STATE

We wanted the response speed of the LU model to be roughly independent of ligand concentration once the system reached its adapted steady-state activity $\overset{‾}{A}=A^{*}(\overset{‾}{c})$. Since the response rate is proportional to sum of the activation and deactivation rates, and ligand concentration enters into the dynamics only through the average receptor state p, this is equivalent to wanting the sum of activation and deactivation rates to have minimal dependence on p when c is held constant.

The sum of activation and deactivation rates is

(C1)
$$k_{\text{response}}\propto \left(1+e^{g(m)}\right)p+\left(1+e^{-g(m)}\right)(1-p).$$

We also know that

(C2)
$$c=\frac{\left(1+e^{g(m)}\right)p}{\left(1+e^{-g(m)}\right)(1-p)}=e^{g(m)}\frac{p}{1-p}.$$

Solving for $e^{g(m)}$ in Eq. (C2) and substituting it into Eq. (C1), we find

(C3)
$$k_{\text{response}}\propto \left(1+\frac{c(1-p)}{p}\right)p+\left(1+\frac{p}{c(1-p)}\right)(1-p),$$

which simplifies to

(C4)
$$k_{\text{response}}\propto c(1-p)+\left(1+\frac{p}{c}\right).$$

If we take the ratio of $k_{\text{response}}$ when p=0 to $k_{\text{response}}$ when p=1, we find that

(C5)
$$\frac{k_{\text{response}}(p=0)}{k_{\text{response}}(p=1)}=c\text{.}$$

That means that, for different values of p, and thus ligand concentration, the response rate can change by at most a factor of c. Since, as discussed in the main text, $\overset{‾}{c}$ is should always be close to 1, the response rate after adaptation should be very close to independent of the ligand concentration adapted to.

## APPENDIX D: STEADY-STATE ACTIVITY AND APPROXIMATION

The mean-field equation for the steady-state activity of the LU model is given by Eq. (A1). This equation is rather unwieldy, however, so we use Eq. (5) to approximate it in the main text when we analyze the origins of large gain. Here we include some additional results supporting the use of the this approximation.

First, we should note that Eq. (5) is exact in the case where $m=m_{0s}$. It is only when $m\neq m_{0s}$ that it becomes an approximation.

To see how the two equations deviated from each other, we compared their predicted dose response curves for various values of m [Fig. 8(a)]. As expected, they are in close agreement near $m=m_{0s}$ and begin to differ only when m changes significantly.

Because we use Eq. (5) primarily to understand the origins of gain in the LU model, we wanted to specifically check that the gain is well represented by the approximation over a range of k and m values. From both approximate and exact dose response curves, we calculate the Hill coefficients,

(D1)
$$h=\frac{{\text{log}}_{10}(81)}{{\text{log}}_{10}\left(\left[L_{90}\right]/\left[L_{10}\right]\right)},$$

where $\left[L_{90}\right]$ and $\left[L_{10}\right]$ are the ligand concentrations at which a given dose response curve reached 90% and 10% of their individual maximum activity. The Hill coefficients show remarkable agreement between the approximation and the exact equations, even for m far from $m_{0}$ [Figs. 8(b)–8(d)].

## APPENDIX E: MEAN-FIELD SUSCEPTIBILITY

The equations for the mean-field susceptibility for the Ising model were taken from Ref. [61].

To calculate the mean-field susceptibility, we start from from the modified steady-state activity used earlier [Eq. (5)]:

(E1)
$$A^{*}=\frac{k(c+1)+1-c-\sqrt{\left[k(c+1)+1-c{]}^{2}-4ck(1-c\right)}}{2(1-c)}.$$

We’re interested in calculating $\chi =dA^{*}/{\left.dc\right|}_{c=1}$. Because the denominator of Eq. (5) goes to zero when c=1, we’ll evaluate χ by using a Taylor expansion of $A^{*}$ in terms of ϵ=c-1. To first order ϵ, we find

(E2)
$$A^{*}=\frac{1}{2}+\epsilon \left(\frac{1}{8k}+\frac{1}{4}\right).$$

Then

(E3)
$$\chi =\frac{dA}{dc}=\frac{dA}{d\epsilon }=\frac{1}{8k}+\frac{1}{4}.$$
